# Acute Pancreatitis Presenting as Inferior Wall Myocardial Ischaemia: A Case Report

**DOI:** 10.7759/cureus.91544

**Published:** 2025-09-03

**Authors:** Inês Ferreira, Inês Fiúza M. Rua, Diogo Ramos, Sérgio Cabaço, André Valente

**Affiliations:** 1 Internal Medicine, Unidade Local de Saúde São José, Lisbon, PRT

**Keywords:** acute coronary syndrome, coronary angioplasty, necrohemorrhagic pancreatitis, pancreatitis, st elevation myocardial infarction

## Abstract

Acute pancreatitis usually presents with epigastric or diffuse abdominal pain. Due to the proximity of the pancreas to the heart, several electrocardiographic abnormalities have been reported. Although ST-segment depression is one of the most commonly reported alterations, localized ST-segment elevation mimicking an acute coronary syndrome (ACS) has only been reported in a few cases. We present a 92-year-old male patient with epigastric pain, whose electrocardiogram (ECG) was concerning for inferior wall ST-elevation myocardial infarction (STEMI) and who was admitted for angioplasty. The final diagnosis after complete blood work was stable coronary artery disease, and the patient was found to present with underlying necrohaemorrhagic pancreatitis. The patient was treated accordingly, with intravenous fluids, vasopressors and antibiotic coverage, but unfortunately died 48 h after admission. This case highlights the rarity and difficulty in discerning a true inferior wall STEMI and a pancreatitis mimicking the ACS and underscores the importance of reporting such cases to raise clinical awareness and guide appropriate management.

## Introduction

Acute pancreatitis (AP) can rarely present with electrocardiographic changes that mimic an inferior wall ST-segment elevation myocardial infarction, posing a significant diagnostic challenge for clinicians. AP is often a mild disease, resolving itself in most patients with an overall in-hospital mortality below 5% [1], and it typically presents with epigastric or diffuse abdominal pain, nausea and vomiting [2]. It has also been reported to present with several electrocardiographic abnormalities, most commonly T-wave inversion and ST-segment depression [3]. Despite this, the presentation of pancreatitis with ECG mimicking an inferior wall ST-segment elevation myocardial infarction (STEMI), with elevation of the ST-segment in contiguous leads II, III and aVF, is described only in a few rare cases, with two published reviews accounting for less than 40 cases each [4,5]. It is also relevant to note that the electrocardiogram (ECG) is one of the most sensitive and specific exams in the diagnosis of STEMI, with a sensitivity of 81%, a specificity of 69% and a positive predictive value of 72% [6]. Contrary to pancreatitis, which presents with a mortality of 1-5% overall [7], STEMI has a higher mortality. Different studies have documented a one-year mortality between 8.4% and 10% due to cardiac causes [8,9] even after successful percutaneous coronary intervention (PCI).

The saying "don't judge a book by its cover" may be applied to several medical diagnoses, highlighting the importance of correlating the clinical history with laboratory and radiologic findings. One such case is the differential diagnosis of epigastric pain, which may be the presenting symptom of an inferior myocardial infarction or one of many abdominal conditions, such as pancreatitis. We report a case of acute pancreatitis presenting with ECG changes mimicking inferior STEMI in a patient with stable coronary artery disease, with negative troponins, highlighting the rarity of pancreatitis-induced STEMI patterns in patients with underlying coronary artery disease (CAD) [5].

## Case presentation

**Figure 1:**
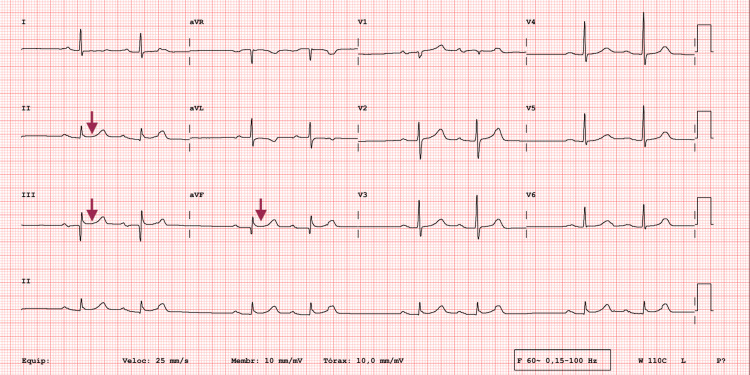
Admission ECG with ST-segment elevation in the inferior leads (II, III, aVF) Admission ECG mimicking an inferior wall STEMI, with ST-segment elevation in the inferior leads as shown by the arrows. STEMI: ST-elevation myocardial infarction.

A 92-year-old male patient with a past medical history of hypertension, dyslipidemia, stable triple-vessel CAD, and obstructive sleep apnea presented to the emergency department (ED) with sudden-onset epigastric pain and nausea that began four hours prior to admission. He was fully autonomous in his daily activities and showed no signs of dementia. On initial examination by the surgical team, the abdomen was tender in the epigastric region without signs of peritoneal irritation. The patient was fully conscious and oriented, with stable vital signs: blood pressure 161/89 mmHg and heart rate 59 beats per minute. An electrocardiogram performed on admission (Figure 1) demonstrated ST-segment elevation and a pathological Q wave in the inferior leads (II, III, and aVF). Initial laboratory studies (Table 1) revealed leukocytosis with neutrophilia, while C-reactive protein remained within normal limits.

**Table 1:** Significant altered laboratory parameters upon ER admission

Laboratory parameter	Patient’s value	Reference range
Leukocytes	14.89x10^9^/L	4.5-11.0x10^9^/L
Neutrophils	12.49x10^9^/L	2.0-8.5x10^9^/L
C-reactive protein	1.6 mg/L	<5.0 mg/L
Arterial pH	7.56	7.35-7.45
Arterial CO_2_ partial pressure	23.8 mmHg	35-45 mmHg
Arterial lactate levels	2.5 mmol/L	<1 mmol/L

Given the ECG findings suggestive of an inferior wall STEMI, the patient was promptly taken for emergent PCI while awaiting troponin results. Coronary angiography confirmed the previously known three-vessel CAD, and the right coronary artery obstruction was treated with angioplasty and stent placement. Subsequent laboratory evaluation (Table 2) revealed negative troponin levels, suggesting stable CAD and therefore no acute coronary syndrome. Markedly elevated pancreatic amylase and lipase levels pointed toward a diagnosis of acute pancreatitis. The patient was subsequently admitted to a level two intensive care unit for close monitoring.

**Table 2:** Complete laboratory parameters upon admission

Laboratory parameter	Patient’s value	Reference range
Pancreatic amylase	1773 U/L	13.00-53.0 U/L
Lipase	4133 U/L	13.0-60.0 U/L
Lactate dehydrogenase (LDH)	787 U/L	135-225 U/L
C-reactive protein	269.7 mg/L	<5.0 mg/L
Glucose	228 mg/dL	60-100 mg/dL
Urea	40 mg/dL	16.6-48.5 md/gL
Creatinine	1.16 mg/dL	0.67-1.17 mg/dL
Troponin T (high sensitivity)	9.1 ng/L	<14 ng/L

**Figure 2:**
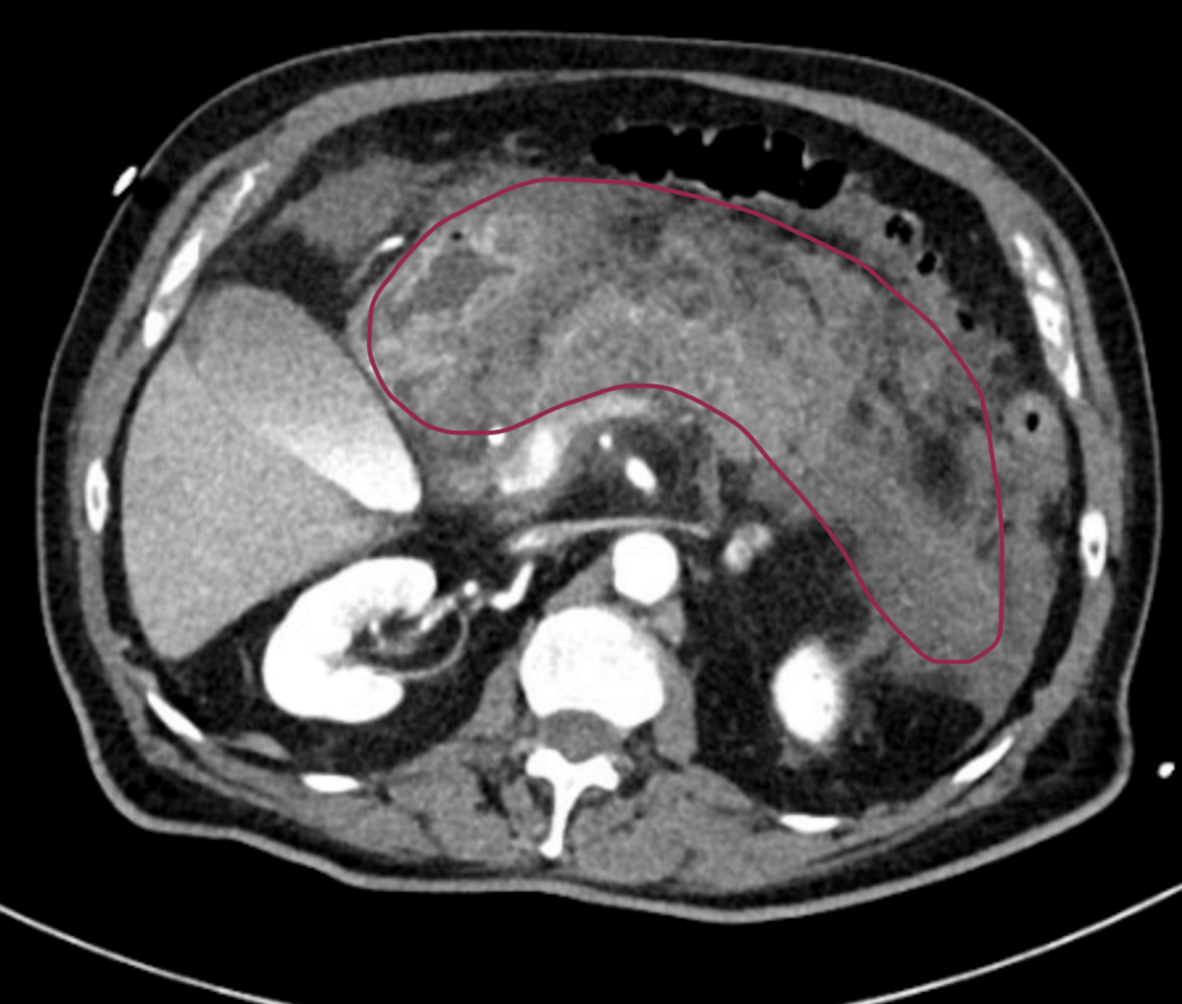
Abdominal CT scan showing extensive necrohemorrhagic pancreatitis Abdominal CT scan showing necrohemorrhagic pancreatitis, as shown by lined delimitation, requested after clinical worsening of the patient.

During hospitalisation, due to progressive aggravation of pain and intolerance to oral intake, accompanied by hypotension and diminishing urinary output, the patient was started on vasopressors. An emergent abdominal computed tomography (CT) scan was requested, showing extensive necrohemorrhagic pancreatitis with multiple fluid collections (Figure 2). General Surgery consultation recommended conservative management, considering the risks of surgery in an elderly patient with significant heart disease.

Repeated troponin dosing was negative (Table 3), reinforcing the exclusion of an acute myocardial infarction as a diagnosis. Post-PCI echocardiography demonstrated left ventricular wall hypertrophy with mild hypokinesia of the inferior septum and no pericardial effusion. Review of previous ECGs, which were not available at the time of admission, confirmed that the pathological Q wave in the inferior leads was pre-existing and that there had been no prior ST-segment elevation.

**Table 3:** Serial troponin dosings during hospitalization PCI: Percutaneous coronary intervention.

	Admission	Post-PCI	Day 1	Day 2
Troponin T (high sensitivity)	9.1 ng/L	8.1 ng/L	13.8 ng/L	11.3 ng/L

Despite aggressive fluid resuscitation and vasopressor support with noradrenaline, the patient’s condition worsened progressively. His pancreatitis was classified as severe, with a Ranson score of 3 (predicted mortality 15%), a Bedside Index for Severity in Acute Pancreatitis (BISAP) score of 2, and a Balthazar CT severity index of 8. Unfortunately, he died 48 hours after admission.

## Discussion

Acute pancreatitis is a relatively common finding in the ED, usually presenting with epigastric pain, nausea, vomiting, elevated pancreatic amylase and lipase, leukocytosis and elevated C-reactive protein (CRP) [2]. Although pancreatitis may present with several ECG abnormalities, this case highlights the possibility of inferior pericardial irritation masquerading as STEMI, which has been described in patients without coronary disease but can also be present in patients with stable disease such as this one [4]. The pathological mechanism by which the ST segment elevation occurs is not clear; several theories have been proposed, such as serum electrolyte alterations, direct myocyte damage by pancreatic enzymes, and hypovolemia leading to low coronary perfusion. These remain as theories and have not yet been clarified. So far, there is also no relation identified between the levels of amylase, lipase and the ECG alterations [10].

While pancreatitis usually has a non-invasive approach on admission, with administration of fluids and analgesia, the prognosis of STEMI depends on the duration of ischaemia, and the guidelines are clear that the ideal time for angioplasty is 60-90 minutes after first medical contact, because each 10 minutes of delay after ischaemia will increase mortality [11,12]. Although the diagnosis of STEMI, according to the American College of Cardiology (ACC) and the American Heart Association (AHA) 2025 guidelines [11], is based on both clinical history, symptoms, ECG and troponin dosing, each hospital may have different laboratory methods for the parameter. In our hospital, the mainstay treatment for STEMI is PCI and it is sometimes performed before troponin dosing is available, when the ECG is highly suggestive, in order to reduce the time between first medical contact and PCI. 

Our patient presents with several singularities that complicate the case further. It is relevant to note that he was on chronic therapy with rivaroxaban for atrial fibrillation, which has been shown to raise the risk of progression from inflammatory pancreatitis to the hemorrhagic form [13]. It is also relevant to note that, although echocardiogram was not performed prior to PCI in order to rule out wall motion abnormalities, he had a previously known triple vessel CAD, and a previously known pathological Q wave in the inferior leads, suggesting a previous inferior wall infarction. Therefore, the echocardiogram could have been inconclusive and Cardiology opted for emergent PCI without previous cardiac imaging, which was only performed after PCI. No previous echocardiograms were available.

This highlights the importance of a thorough clinical and analytical evaluation before establishing a definitive diagnosis, especially when one of the two diagnostic hypotheses is potentially life-threatening and may need immediate care. Although 34 cases of acute pancreatitis mimicking inferior wall STEMI have been reported in the literature [4], continued reporting is essential to raise awareness of this rare presentation, facilitate earlier recognition, improve patient management, and eventually assemble sufficient data for statistically meaningful conclusions.

## Conclusions

This case illustrates one of medicine’s more challenging diagnostic dilemmas: distinguishing between STEMI and other conditions that can present with ST-segment elevation, such as acute pancreatitis. Both entities demand a high index of suspicion and usually require more than one diagnostic modality to reach a definitive diagnosis. The overlap of symptoms and ECG abnormalities, as seen in this patient, further complicates timely differentiation. This case underscores the importance of integrating a thorough medical history, comprehensive laboratory evaluation, and abdominal imaging when clinical doubt persists. Given the rarity of acute pancreatitis mimicking inferior wall STEMI - with only 34 cases reported in the literature - continued case reporting remains vital to increase awareness, improve recognition, and eventually provide sufficient data to guide evidence-based decision-making. The more cases are documented, the greater the likelihood of identifying distinguishing clinical, laboratory, or imaging features that could aid in differentiating the two diagnoses upon admission or even prior to PCI, thereby balancing the need for urgent reperfusion with diagnostic accuracy. Finally, this growing body of evidence may one day enable the development of structured diagnostic pathways or clinical decision tools designed to reduce unnecessary angiography, while preserving the imperative of rapid reperfusion in true STEMI.
